# White Graphene-Cobalt Oxide Hybrid Filler Reinforced Polystyrene Nanofibers for Selective Oil Absorption

**DOI:** 10.3390/polym12010004

**Published:** 2019-12-18

**Authors:** Deepalekshmi Ponnamma, Sabari S Nair, Hemalatha Parangusan, Mohammad K. Hassan, Samer Adham, Alamgir Karim, Mariam Al Ali Al-Maadeed

**Affiliations:** 1 Center for Advanced Materials, Qatar University, Doha P.O. Box 2713, Qatar; sabari.nair@qu.edu.qa (S.S.N.); hemakavin@gmail.com (H.P.); mohamed.hassan@qu.edu.qa (M.K.H.); m.alali@qu.edu.qa (M.A.A.A.-M.); 2 ConocoPhilips Global Water Sustainability Center, Qatar Science and Technology Park, Doha 24750, Qatar; Samer.Adham@conocophillips.com; 3 Department of Chemical & Biomolecular Engineering, University of Houston, S222 Engineering Bldg 1, 4726 Calhoun Rd, Houston, TX 77204, USA; akarim3@Central.UH.EDU; 4 Materials Science and Technology Program, Qatar University, Doha P.O. Box 2713, Qatar

**Keywords:** hydrophobicity, co-precipitation, oil absorbent, nanofibers

## Abstract

In this work, stable hydrophobic nanocomposites are made from electrospun fibers of polystyrene (PS) containing a hybrid filler combination of (i) hexagonal boron nitride (hBN) and (ii) cobalt oxide (Co_3_O_4_) nanomaterials. Good synergistic interaction is observed between the nanomaterials, since the growth of Co_3_O_4_ was carried out in presence of white graphene nanosheets. Filler synergy modifies the PS surfaces, by enhancing the filler-polymer interfacial interactions and provides good tensile strength. The hydrophobic films are gamma irradiated to improve crosslinking within the polymer nanocomposites. Since gamma irradiation enhances the surface roughness, its hydrophobicity/oleophilicity increases much and the final nanofibers show good oil-water separation efficiency. The nanofibers act as sponge clothing to skim the oil from a mixture of oil and water. Durability of the fibers in hot water and in presence of ultrasonic waves is also tested and good response is achieved. Contact angle studies are performed to investigate the surface properties and to check the influence of gamma irradiation on the surface wettability. The gamma-irradiated PS nanocomposite fiber shows a contact angle of 152° ± 2° compared to the 140° ± 1° of the neat PS fiber, evidencing the superhydrophobicity. Both the effects of crosslink density enhancement and hybrid filler distribution make the composite fibers stronger in oil absorption application even at higher operation temperatures. The fibers are reported to be robust and durable, in addition.

## 1. Introduction

Super enhancing manufacturing industries and frequent oil spills, along with the waste disposal to water bodies, are polluting the aquatic environment at an alarming rate [1,2,3]. Chances of catastrophic oil spills, especially in the Gulf region, is a matter of utmost priority, and the possible solution of oil removal from water surfaces by an efficient and cost effective way is highly desired [4,5]. In this regard, flexible polymer-based membranes are attracting huge research interest because of their selective super wetting properties, low cost, tunable surface structure, and various designing possibilities [5,6]. Polymeric nanofiber membranes are fabricated by the most versatile electrospinning technique, by which the fiber diameter can be tailored from a few nanometers to micrometer [5,7,8]. The interconnected small pores, good wettability, uniform fiber diameter, and high flexibility are the most significant features of electrospun fibers that make them useful in oil-water separation. Filtration efficiency depends on the fiber diameter and many polymers are being tested for their possibility in separating oil-water emulsions through their electrospun fiber mats [6,9]. Fibers of controlled structures and compositions are made by reinforcing the polymer with nanofillers so that unique physical, chemical, and mechanical properties are targeted for the synthesized membranes. The superior nanostructure technology can be combined with biomimetic strategy to create advanced surface wettability and functionalities [10]. Since nanoarchitectures influence the surface compositions to very great extents, superhydrophobic membranes made from the nanostructures are also becoming an advisable choice [11,12]. 

Electrospun polystyrene (PS) fibrous sorbents were synthesized by Wu et al. to investigate their sorption capacity towards four different oils—peanut oil, motor oil, diesel oil, and silicon oil [7]. Highest adsorption capacity of 100 g oil/1 g of the sorbent was achieved for the prepared super hydrophobic and super oleophilic fibers. Similarly, Lee et al. fabricated PS nanofibers capable of separating low viscous oil from water, with no efficiency of separation for the highly viscous oil [2]. Since PS is a commercially significant polymer, its nanofibers are supposed to possess good hydrophobicity (up to 160°) and good mechanical properties [13]. As the surface wettability of a typical film depends on the chemical composition and topography, hydrophobic/oleophilic properties are applied to tune porous surfaces for achieving enough roughness. PS-based nanofibers are emerged as good choices for effective oil-water separation, especially when modified nanomaterials are embedded within [14,15,16].

Hexagonal boron nitride or hBN (white graphene) [17] is a dielectric and thermally conducting filler, which can enhance the dielectric, mechanical, and thermal properties of a polymer. In addition, its two dimensional structure can influence the porous structure of the electrospun polymer composites and also can influence the migration pathways of oil-water molecules during oil-water separation [18,19]. Structurally stable hBN is chemically inert, oxidation resistant, and has an average thermal conductivity of 30 Wm^−1^K^−1^ [17]. This material is also used in fabricating hydrophobic membranes and in filtration systems. It also has significance in cosmetics due to the oil and IR absorbing capability. Chemically stable materials, like hBN, incorporation is necessary to treat industrial problems related to oil spills [19,20]. This will help to enhance the durability of the manufactured membranes. It is also reported that the boron nitride nanotubes aligned in specific directions enhanced the surface roughness and non-wettability [21]. However, in hBN-filled polymers, the filler-polymer interfacial interaction and dispersibility are major issues to be considered and proper surface modification (chemical/physical) is necessary to achieve maximum efficiency for nanocomposite properties [15,16,17]. 

Transition elements in group VIII and IB and their oxides are well known for their strong interaction with the polar groups, thus enhancing the surface coating interactions [11,22]. More specifically, the spinel cobalt oxide (Co_3_O_4_) is notable for its p-type semiconducting nature and it possesses a typical porous structure, depending on the synthesis route [23]. Addition of this filler to polymer enhances the porosity of the nanocomposites, in addition to good flexibility, softness, and air permeability [24]. The sufficient surface area of the polymer fibers also helps to bond with metal oxides by polar group absorption [22]. 

Since the synergistic combination of fillers always increase the rate of dispersion of both fillers in the polymer matrix [25,26,27,28,29], the combination of hBN and Co_3_O_4_ is done here in this work, to fabricate the nanocomposite fibers of PS. Both the polymer and the composites are fabricated by electrospinning and followed by gamma irradiation to trigger crosslink formation; and thus to enhance the surface roughness. This helps to create enough pores and to enhance the surface hydrophobicity. The hydrophobic nanofibers are tested for the oil-water separation experiments, which revealed the oil absorbing nature of the fibers. Mechanically strong fibers of the hybrid filler composite were able to maintain the durability in hot water (up to 80 °C) and also when subjected to ultrasonication.

## 2. Experimental Details

### 2.1. Materials

Cobalt (II) chloride 6-hydrate, N,N-dimethylformamide (DMF) solvent and the PS pellets (M_W_~280,000) were purchased from Sigma-Aldrich (St. Louis, MI, USA). NaOH and Isopropyl alcohol (IPA) were obtained from VWR Prolabo Chemicals (Batavia, USA). The hexagonal boron nitride (hBN) of average particle size 50 nm was purchased from Sisco Research Laboratories, PVT Ltd (Mumbai, India). 

### 2.2. Synthesis of Cobalt Oxide

Co_3_O_4_ nanoparticles were synthesized following the co-precipitation method [30]. For this, around 2 g Cobalt (II) chloride 6-hydrate was dissolved in 50 mL distilled water, using magnetic stirring, followed by the addition of 1.2 g NaOH in 10 mL water. Further, 5 mL IPA was added dropwise and the whole mixture was stirred for 1 h at 70 °C and kept under stirring overnight at room temperature. The precipitate obtained was filtered, washed with water until neutrality was achieved, and thereafter dried at 120 °C for 3 h. The obtained powder was finely ground and then calcined in the tube furnace at 400 °C for 2 h. The final powder sample was kept for the analysis.

### 2.3. Synthesis of hBN/Co_3_O_4_ Hybrid Nanofiller

The hybrid hBN combined with Co_3_O_4_ nanomaterials were synthesized following the same preparation method of Co_3_O_4_ in presence of hBN. More clearly, 2 g cobalt (II) chloride 6-hydrate dissolved in 50 mL distilled water under magnetic stirring and 0.2 g hBN powder ultrasonicated in 5 mL IPA for 60 min were prepared separately and thereafter mixed together. The NaOH (1.2 g in 10 mL water) solution was then added and the whole mixture was stirred for 1 h at 70 °C using magnetic stirring. The mixture was kept for stirring overnight at room temperature, and the precipitate obtained was washed, filtered, and dried at 120 °C for 3 h. Calcination at 400 °C for 2 h was also done for the ground nanomaterial and finally kept for further characterization. 

### 2.4. Synthesis of PS Nanocomposites by Electrospinning 

PS nanocomposites containing hBN, Co_3_O_4_ and hBN/Co_3_O_4_ were prepared through electrospinning technique. First the PS pellets (2 g) were dissolved in DMF (6 mL) at 50 °C using magnetic stirring. Specific amounts of Co_3_O_4_, hBN, and hBN/Co_3_O_4_ (to obtain 1 wt.% filler composite) were dissolved separately in 4 mL DMF by ultrasonication for 60 min. The ultrasonication helped to exfoliate the nanoparticle clusters and enhanced the dispersion. To each PS dissolutions, the individual nanomaterial dispersions were added and magnetically stirred for 24 h to achieve better filler polymer interactions. Those nanocomposite suspensions were electrospun with the following conditions: tip to rotating drum collector distance 12 cm, applied voltage 13 kV, and flow rate 1.5 mL/min. Finally, electrospun nanofibers of pure PS, PS/Co_3_O_4_ at 1 wt.% (PS/Co-O), PS/hBN at 1 wt.% (PS/hB), and PS/hBN/Co_3_O_4_ at 1 wt.% (PS/hBCo-O) were obtained. Then all composite fibers were gamma irradiated at 13 kGy for 24 h using Cobalt-60 high dose irradiation from MDS Nordion International (Nordion Inc., Ottawa, ON, Canada). 

### 2.5. Characterization Techniques

Morphological characteristics of the synthesized samples (nanopowders and the nanocomposite fibers) were studied using field emission scanning electron microscope (Nova Nano FESEM, Thermo Fisher Scientific, Waltham, MA, USA) at an accelerating voltage of 5.0 kV and transmission electron microscope (TEM, FEI TECNAI GF20 S-TWIN). Nanopowder samples for the TEM analysis were prepared by dispersing them in ethanol and dropping the dispersants onto carbon copper grids. Similarly, the fibrous samples were made by directly spinning the fibers on copper grids. Structural and phase analysis of the Co_3_O_4_ and Co_3_O_4_/hBN powders along with the composite fibers were performed by X-ray diffraction analysis (PAN analytical X’pert Pro, Almelo, Netherlands) with a scanning rate of 2 °C/min and a scanning angle ranging between 10° ≤ ɵ ≤ 90°) and Fourier transform infrared (FT-IR) spectral analysis (FTIR Thermo Fisher Scientific, Waltham, MA, USA) during the range of 4000 to 500 cm^−1^. X-ray photoelectron spectrometer, Kratos Axis ultra DLD (Kratos Analytical Ltd., Manchester, UK), was used to conduct high resolution X-ray photoelectron spectroscopy (XPS) measurements. Mechanical properties and tensile strength of the polymer nanocomposites were tested by universal testing machine (Lloyd 1KN LF Plus, AMETEK, Inc., Bognor Regis, UK) at 5 mm/min. Contact angle measurements were done by a drop shape analysis system (OCA 35-Dataphysics, Filderstadt, Germany) using deionized water. Finally, the oil absorption capacity of PS/Co_3_O_4_/hBN composites were compared by filtering a mixture of oil in water emulsion through a vacuum filtration set up.

## 3. Results and Discussion

### 3.1. Morphology and Structural Analysis of the Synthesized Nanomaterials

**Figure 1:**
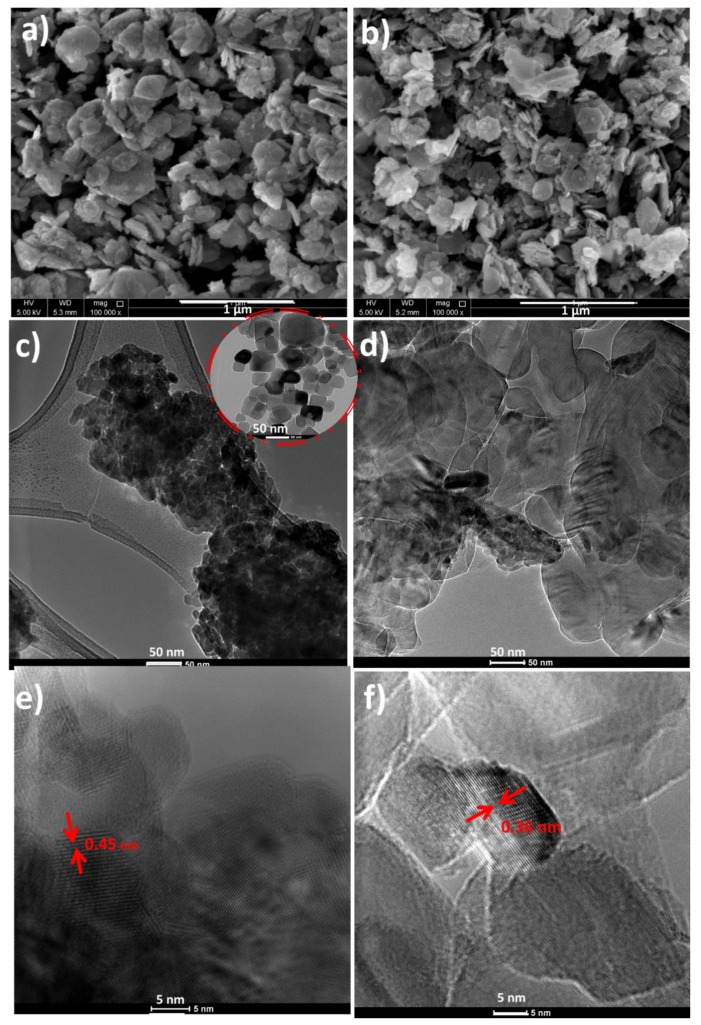
SEM images of (**a**) Co_3_O_4_ and (**b**) hBN/Co_3_O_4_; TEM images of (**c**) Co_3_O_4_ and (**d**) hBN/Co_3_O_4_; and HRTEM images (magnification 700 k) of (**e**) Co_3_O_4_ and (**f**) hBN/Co_3_O_4_. Inset of Figure 1c shows the TEM image of hBN.

The morphology of the synthesized Co_3_O_4_ and hBN/Co_3_O_4_ nanomaterials were examined by the SEM and TEM images, as represented in Figure 1. An aggregated morphology is obtained for the Co_3_O_4_ (Figure 1a), while a hexagonal sheet mixed morphology is seen for the hybrid filler hBN/Co_3_O_4_ (Figure 1b) as expected. Co_3_O_4_ shows an irregular hexagonal nanoplatelet fragment morphology similar to broken disc, as reported elsewhere [31]. This is attributed to the fact that oxidation trigger the breaking of particles [32]. The detailed morphology analysis of the nanomaterials by TEM illustrate interesting facts. As shown in Figure 1c, the Co_3_O_4_ nanomaterials possess a grape bunch morphology, substantiating its cluster formation tendency, and irregular platelet like architecture, in consistent with the SEM results. Figure 1d illustrates the hybrid filler morphology, in which hexagonal boron nitride sheets (inset of Figure 1c) are visible on the Co_3_O_4_ background. 

The lattice parameters and crystalline structural regions can be identified by means of high resolution TEM (HRTEM) images. The HRTEM images for the synthesized Co_3_O_4_ and hBN/Co_3_O_4_ hybrid nanofillers are represented in Figure 1e,f, respectively. Well defined lattice is seen for the Co_3_O_4_, with a distance of 0.45 nm corresponding to the face-centered cubic cobalt oxide Co_3_O_4_ phase [33]. In the case of hybrid filler, the measured lattice spacing (0.36 nm) was smaller than that of the Co_3_O_4_, suggesting that the crystalline regions underwent some distortions during the hybrid filler combination [34]. 

**Figure 2:**
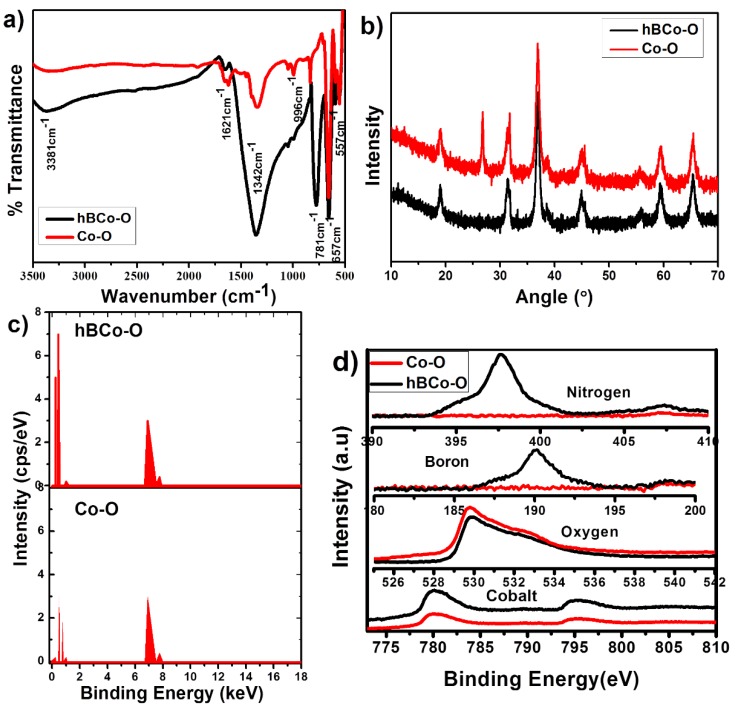
Characterization of Co_3_O_4_ and hBN/Co_3_O_4_ (hBCo-O) nanomaterials: (**a**) FTIR spectra; (**b**) XRD patterns; (**c**) EDAX analysis; (**d**) XPS spectra.

Structural details of the synthesized fillers were studied by FTIR, XRD, EDAX, and XPS analyses. The FTIR spectrum of the synthesized Co_3_O_4_ nano particles is provided in Figure 2a. The spectrum showed the presence of two distinct peaks at 557 and 657 cm^−1^, which were originated from the stretching vibrations of the metal-oxygen bond and confirms the formation of Co_3_O_4_ metallic oxide [35]. More specifically, the band formed at 557 cm^−1^ is characteristic of Co^3+^ vibration in the octahedral hole, while the band at 657 cm^−1^ is attributed to Co^2+^ vibration in the tetrahedral hole in the Co_3_O_4_ spinel lattice. At 657 cm^−1^, the bridging vibration of O-Co-O can also happen. While the band at 1621 cm^-1^ is assigned to the O-H (from the hydroxide still remaining) stretching and bending modes of vibration, the bands at ∼1045 and ∼996 cm^−1^, respectively, correspond to C-H and O-H stretching vibrations. Compared to the Co_3_O_4_, the hybrid nanomaterial showed the appearance of some additional bands, as the one at 1342 cm^−1^ corresponds to the in-plane B-N stretching vibrations, while the band formed at 778 cm^−1^ is due to the out-of-plane B-N-B bending vibrations. In addition, the broad peak around 3337.6 cm^−1^ can be due to the B-OH bonding, which suggests the interaction of Co_3_O_4_ with the hBN nanolayers. 

The crystal structure and phase analysis of the nanomaterials are further analyzed by XRD, as shown in Figure 2b. The spectra reveal the highly crystalline nature of Co_3_O_4_ and hBN/Co_3_O_4_ nanomaterials. The peaks obtained around 18°, 30°, 37°, 45°, 55°, 60°, and 65° of two theta values correspond to the various crystalline planes of (111), (220), (311), (400), (422), (511), and (440) of Co_3_O_4_, respectively [35]. This can be perfectly matched with the cubic crystalline structure of the synthesized cobalt oxide. The figures also show that the hybrid nanomaterial follows exactly the same path of XRD pattern as the Co-O. This corresponds to the presence of Co_3_O_4_ in the hBN/Co_3_O_4_ nanomaterial without losing its structural integration. An additional peak observed around 26.68° is attributed to the presence of the BN phase. Similar peak appearance with enhanced intensity for the hybrid material is attributed to the absence of any impurities or structural deformation of the Co_3_O_4_ crystalline material. In correlation with the HRTEM and FTIR studies, XRD also suggests the formation of cubic crystalline Co_3_O_4_ nanoparticles during the Co_3_O_4_ synthesis and its structural identity retention in the hybrid nanomaterial. Though HRTEM reported the distortion chance, bonding or chemical composition was not affected by the hBN incorporation.

The chemical purity and stoichiometry of both Co_3_O_4_ and hBN/Co_3_O_4_ were monitored by EDAX analysis (Figure 2c) and XPS measurements (Figure 2d). Composition of the elements is very clear from the EDAX plots, as B and N are additionally present in hBN/Co_3_O_4_, other than the Co and O elements in Co_3_O_4_. The spectra also show the absence of additional impurities in the prepared nanomaterials, indicating their structural purity [36,37]. The XPS survey spectrum of Co-O and hBN/Co_3_O_4_ shows the presence of Co, and O atoms in both samples, with additional B and N in the hybrid. The Co 2p XPS spectra show two major peaks at 780.0 and 796.2 eV binding energies, respectively, corresponding to Co 2p_3/2_ and Co 2p_1/2_ [38]. Similar peak appearance and intensities suggest the structural integration of Co_3_O_4_ in the hybrid filler case. No elements were doped or removed during the hybrid filler synthesis. Both the morphology and structural information on the nanomaterials suggest the formation of Co_3_O_4_ and its hybrid combination with hBN; and, further, these nanofillers were used to fabricate the PS-based nanofibers.

### 3.2. Morphology and Structural Analysis of the PS Nanocomposite Fibers 

**Figure 3:**
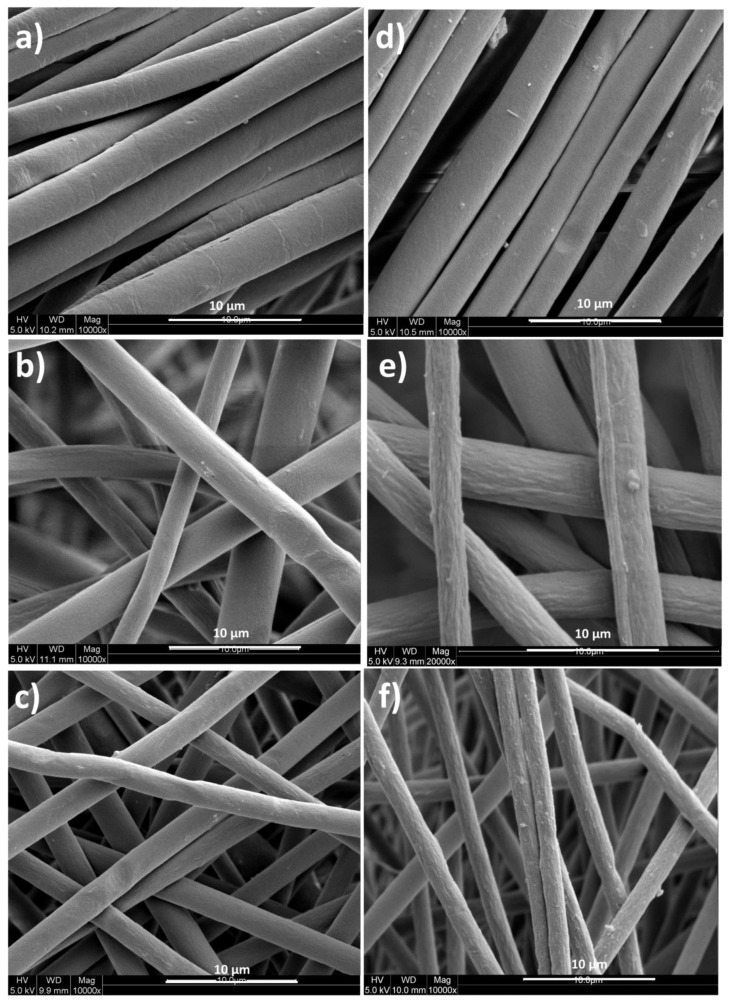
SEM images before gamma irradiation for (**a**) pure PS, (**b**) PS/Co-O, (**c**) PS/hBCo-O; and after gamma irradiation for (**d**) pure PS, (**e**) PS/Co-O; and (**f**) PS/hBCo-O fibers.

Electrospinning typically yields fibers that exhibit one of the three typical morphologies: beads, beads-on-string, and smooth fibers. These different structures can be obtained by adjusting the process parameters and the operation conditions. Here, the fibers are observed with SEM before and after gamma irradiation treatment (Figure 3). Figure 3a,d, respectively, show the pure PS fibers before and after gamma irradiation. This image is taken as a base to understand the effect of the gamma irradiation and to verify the influence of different nanomaterials embedment in the composites. When the images for all samples before and after gamma irradiation are considered, the surface roughness is found to be affected by the radiation. In all cases, after the gamma irradiation the surface became rougher, indicating the chain scission/crosslinking usually associated with gamma irradiation [39,40]. In addition, the fiber diameter also changes upon the addition of nanoparticles. It was found that the nanoparticles in 1 wt.% was able to decrease the average fiber diameter from 2.5 to <1 μm, respectively, from the pure PS to the PS containing hBN and Co_3_O_4_ hybrid fillers. With γ-irradiation, the fibers maintain similar diameter (average fiber diameter after irradiating PS/hBCo-O with γ-rays is 0.97 µm), confirming that there is no influence of irradiation on the fiber diameter. 

**Figure 4:**
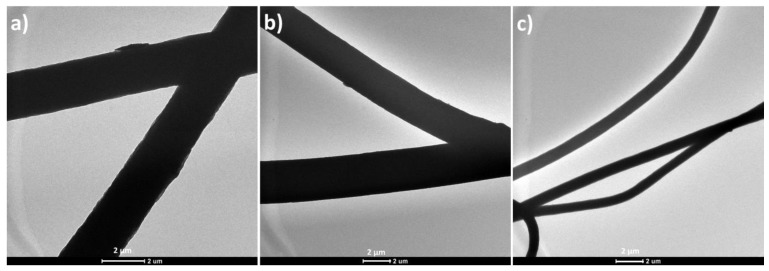
TEM images of (**a**) pure PS, (**b**) PS/Co-O, and (**c**) PS/hBCo-O composites after gamma irradiation.

The fiber formation was further tested by analyzing the TEM images of all PS-based samples. Figure 4 shows the TEM images of the pure PS, PS/Co-O, and PS/hBCo-O samples after gamma irradiation. The change in fiber diameter from μm to nm is clearly seen from the figure. This reduction in fiber diameter due to the incorporation of nanomaterials can be due to the improved interaction of PS chains with the nanomaterial surface and also the reduced surface tension [41]. The nanoparticles are embedded within the polymer chains and not observed on the surface of nanofibers.

**Figure 5:**
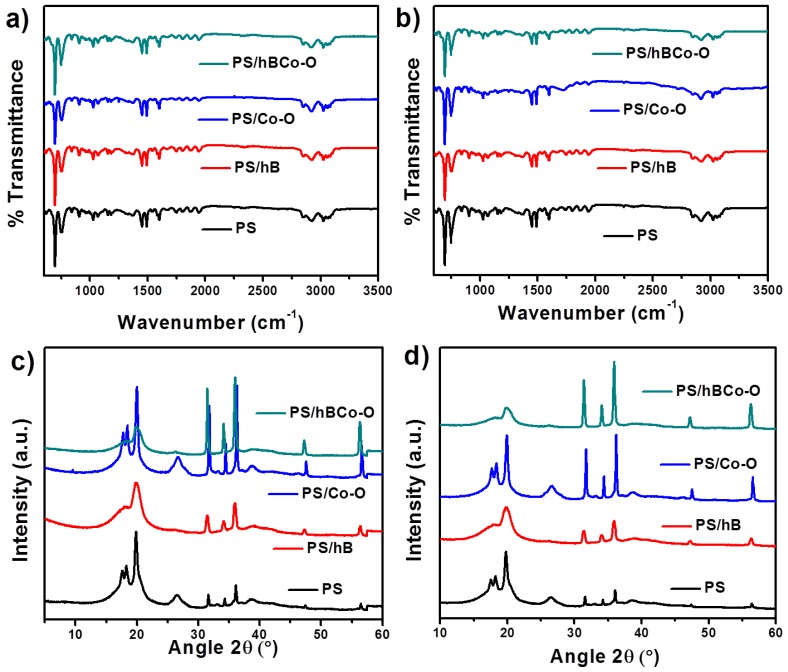
FTIR spectra for the nanocomposites (**a**) before and (**b**) after gamma irradiation. XRD patterns (**c**) before and (**d**) after gamma irradiation.

FTIR and XRD spectral studies of all samples before and after gamma irradiation were done as shown in Figure 5. No significant change was observed in the spectral pattern indicating that the gamma irradiation does not influence the structural integrity of the samples. 

### 3.3. Mechanical Properties of the PS Nanocomposite Fibers 

The difference in mechanical properties of the prepared composites before and after gamma irradiation was analyzed in detail. Table 1 shows the tensile strength, Young’s modulus, and elongation at break values for all the nanocomposite fibers. The mechanical properties of the nanofibers are closely related to the intrinsic strength of the fibers and the bonding among them. It is clear from the table that both tensile strength and Young’s modulus values for the sample containing hBN and Co_3_O_4_ were higher when compared to the individual nanocomposites. In addition, the exposure to gamma irradiation enhanced the mechanical properties corresponding to the bond strengthening and crosslinking. 

### 3.4. Surface Wettability of the Nanocomposite Fibers by Oil and Water 

**Figure 6:**
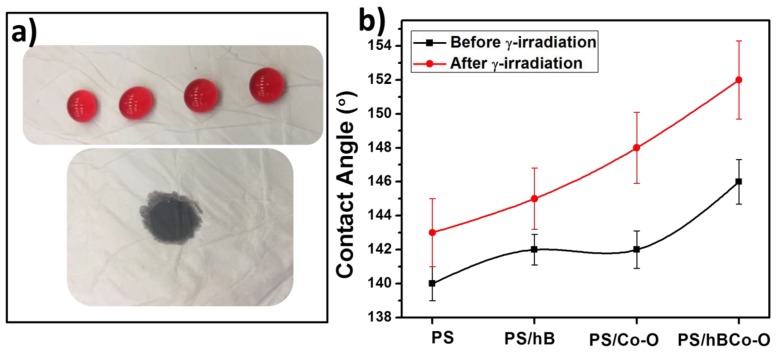
(**a**) Photograph of water droplets and oil droplet on the surface of γ-irradiated PS/hBCo-O composite. (**b**) Contact angle values for the composites before and after gamma irradiation.

In general, the surface free energy for water is higher than that of oil, thus the surface energy of a solid surface is in such a way that both hydrophobicity and oleophilicity can be together achieved [42]. Figure 6a represents the surface wettability of the gamma-irradiated PS electrospun fibers containing hBN and Co_3_O_4_. The figure clearly shows the hydrophobicity and oleophilicity of the fabricated composites, as water droplets rest on the fiber surface and oil molecules penetrate through the fibers. Figure 6b also shows the contact angle images obtained upon wetting the nanofibers with water and oil. The wettability of a surface depends on the surface chemistry and the roughness. Here gamma irradiation enhances the roughness and the irregular hydrophobic surface promotes oil absorption. The incorporation of hybrid fillers and gamma irradiation enhances the oil-water separating efficiency of the nanofibers by increasing the absorption of oil molecules by the fibers allowing water molecules to pass through it.

Other than testing the contact angle values of the composites, oil absorption experiments were carried out by dripping a specific amount of oil on all samples and the efficiency of oil absorption was compared by weighing the composites before and after oil absorption. Table 2 shows the relative fraction of absorbed oil on the fibers. From these values, the influence of gamma irradiation in enhancing oil absorption is clear. When all the composites were considered, the one containing the hybrid filler composition showed the best oil absorption capability. 

**Figure 7:**
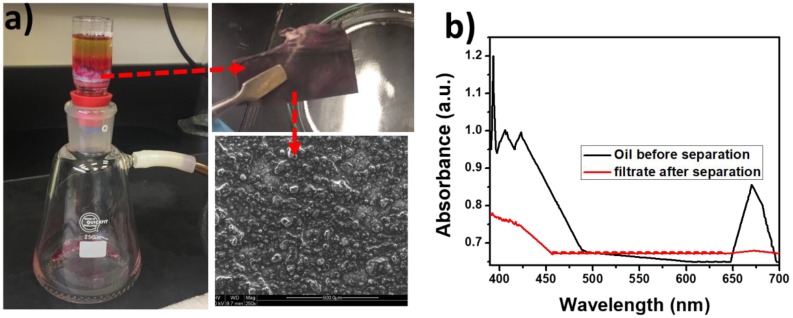
(**a**) Experimental set up used for separating the oil-water mixture, and (**b**) UV-visible absorption spectra for the oil/water mixture before and after the oil absorption.

The experimental set up used for separating oil-water mixture is given in Figure 7a, and the process is further illustrated by the Supplementary Video. Upon pouring a mixture of oil and water in to the Buchner funnel containing the electrospun PS composite fibers underneath, the oil molecules get absorbed on the fibers, allowing the water to permeate through the composite and finally to the flask. Oil remains on the top of the fiber. It is also found that the separation efficiency depends on the amount and nature of oils used. In the typical experiment, a total volume of 30 mL was used for the separation; however, increased oil contents in the mixture delayed the separation process. In addition, the efficiency will be affected when high viscous oils are used [19]. The supporting video clearly illustrates the efficiency of vacuum filtration set up in selectively separating the oil. The oil permeation was not observed even after 30 min of the oil filtration experiment. The photographs given in Figure 7a also clearly explain the complete oil absorption by the nanocomposite fiber. It is also understood from the SEM image of the oil absorbed fiber (inset of Figure 7a), that the oil molecules are trapped in the fiber structure on the surface.

The evidence of oil-water separation was further obtained by the UV-Visible spectra shown in Figure 7b. The filtrate shows no indication of the presence of oil molecules as the peak associated with the oil is not obtained.

The strong interactions between the Co_3_O_4_ and hBN along with the gamma irradiation create a hydrophobic surface for the as prepared nanocomposites. These points, as clear from the morphology and structural analysis of the hybrid nanocomposite, substantiate the oil-water separation process. Such oil absorbed fibers can be cleaned to regenerate the fibers using mild NaOH or surfactant solution. This can be done by rinsing the fibers in typical solvent for a few minutes and, thereafter, drying it.

**Figure 8:**
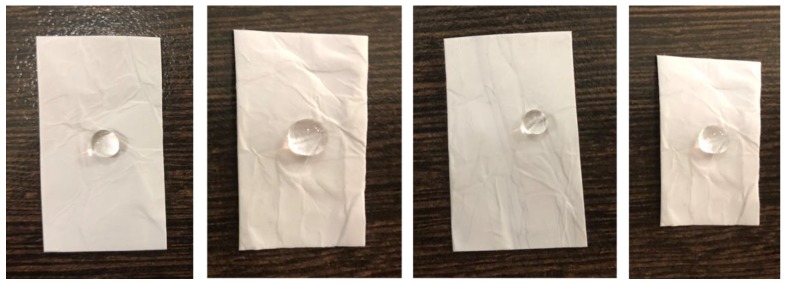
Photographs of water droplets on the PS/hBCo-O gamma-irradiated fiber surface maintained at 20, 40, 60 and 80 °C.

Investigating the durability of the nanocomposite is necessary to broaden its applicability in oil-water separation. Since the surface tension of water decreases with increase in temperature, monitoring oil-water separation at higher temperatures is a matter of high significance. Most of the hydrophobic surfaces show water repellency at low temperature and at higher temperatures the repellency will be lost. The super-hydrophobicity of the water droplets is checked by observing the water droplets on the fiber surface maintained at 20, 40, 60, and 80 °C (Figure 8). It is observed that the shape (uniform contact angle) of the water droplets is unaffected by the temperature indicating the high stability and water repellent behavior of the gamma-irradiated PS/Co_3_O_4_-hBN fibers. 

**Figure 9:**
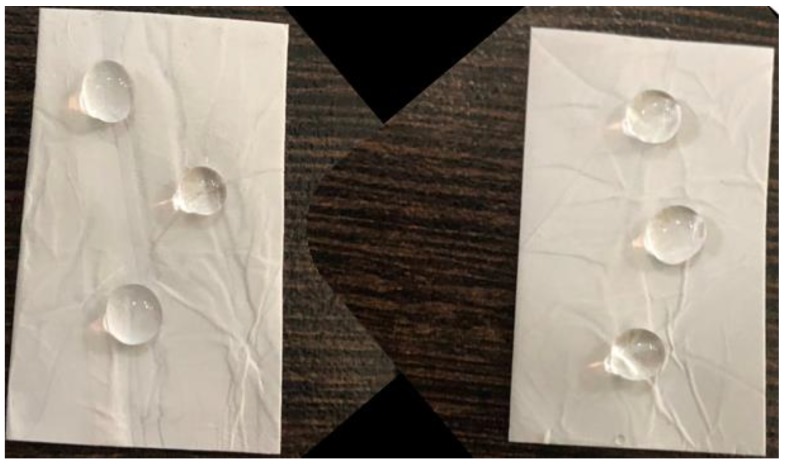
Photographs of water droplets on the PS/hBCo-O gamma-irradiated fiber surface before and after ultrasonication.

The loss of super hydrophobicity over repeated oil absorption or long-term use is a serious problem which negatively affects the oil-water separating efficiency of a typical composite. The investigation of long-term durability of the nanocomposite fibers was done by immersing the nanofibers in aqueous medium and ultrasonicating the fiber for 15 min. The photographs in Figure 9 show the water droplets on the surface of the nanocomposites after ultrasonication, which confirm the durability of the synthesized nanocomposites. This substantiates the use of PS fibers embedded with hybrid nanofillers in smart absorbers for oil in water. 

Finally, in addition to the oil-water separation, multifunctional textile applications can also be targeted for the fabricated composites, to the thermal conductivity of hBN and the magnetic properties of Co_3_O_4_. 

## 4. Conclusions

The hybrid filler combination containing hexagonal sheet-like hBN and grape bunch-like cobalt oxide cubic crystalline nanoparticles were synthesized following the co-precipitation method, and added to PS in 1 wt.%. Nanocomposite fibers made by electrospinning were gamma irradiated so that better crosslinking efficiency and surface roughness were achieved. The mechanical strength and Young’s modulus were enhanced by 1.09% and 1.12%, respectively, for the gamma irradiated composite compared to the non-irradiated ones. The contact angle value of 152 ± 2° for the gamma-irradiated PS/hBN-Cobalt oxide points out the super-hydrophobicity of the composite. Due to this reason it acted like a good oil absorber and also a good fiber platform to separate oil from oil-water mixture. The durability of the film was checked by temperature treatment and ultrasonicating the fibers, and the synergistic composite showed good durability. The composite nanofibers also proved that the temperature has no effect on oil absorption. 

## Figures and Tables

**Table 1:** Mechanical properties of the nanocomposites.

Sample		Tensile Strength (MPa)	Young’s Modulus (MPa)	Elongation at Break (%)
**Non irradiated**	PS	28.54 ± 1.1	72.50 ± 6.44	18.12 ± 1.22
	PS/Co-O	30.47 ± 2.3	76.33 ± 2.21	17.24 ± 1.10
	PS/hBN	35.44 ± 2.5	77.65 ± 4.05	17.39 ± 0.98
	PS/hBCo-O	48.24 ± 2.6	98.15 ± 4.79	10.23 ± 0.77
**Irradiated**	PS	30.87 ± 1.3	73.30 ± 9.01	19.90 ± 1.01
	PS/Co-O	31.05 ± 2.2	77.88 ± 8.75	17.90 ± 1. 12
	PS/hBN	38.25 ± 1.7	82.03 ± 2.45	16.35 ± 2.25
	PS/hBCo-O	52.54 ± 2.1	110.35 ± 4.55	9.50 ± 2.15

**Table 2:** Amount of absorbed oil on the nanofibers.

Sample		Weight before Oil Absorption	Weight after Oil Absorption	Increase in Weight
**Non irradiated**	PS	0.1208	0.8413	0.7205
	PS/Co-O	0.1263	1.1762	1.0499
	PS/hBN	0.0921	1.273	1.1809
	PS/hBCo-O	0.1035	1.3140	1.2105
**Irradiated**	PS	0.0526	0.7761	0.7235
	PS/Co-O	0.1193	1.7546	1.6353
	PS/hBN	0.1244	1.4430	1.3186
	PS/hBCo-O	0.3838	2.592	2.2082

## Supplementary Materials

The following are available online at https://www.mdpi.com/2073-4360/12/1/4/s1, Video S1: Oil/Water Separation using the PS-nanocomposite fibers.
